# Evaluating the value of neoadjuvant radiotherapy in patients with breast cancer

**DOI:** 10.1007/s12672-026-05466-3

**Published:** 2026-06-20

**Authors:** Wenyan Dong, Yao Pan, Zhenglong Wu, Dengfeng Wu

**Affiliations:** Department of Thyroid and Breast Surgery, The Affiliated Wuxi People’s Hospital of Nanjing Medical University, Wuxi Medical Center, Nanjing Medical University, Wuxi, 214023 Jiangsu China

**Keywords:** Neoadjuvant therapy, Radiotherapy, Breast cancer, Overall survival, SEER

## Abstract

**Background:**

The benefits of preoperative neoadjuvant radiotherapy (NART) for breast cancer are not conclusive and there are no well-established guidelines for NART. We aimed to analyze the difference in survival benefit between NART and postoperative adjuvant radiotherapy (PORT) and screening patients who are suitable for NART.

**Methods:**

A retrospective cohort study using the Surveillance, Epidemiology, and End Results (SEER) from 2010 to 2021 was performed. We comprehensively assessed trends in the management of NART in the population during the past decade. The factors associated with NART were then explored using logistic regression. We also compared BC patients with preoperative neoadjuvant radiotherapy (NART) or postoperative adjuvant radiotherapy (PORT) for overall survival (OS) and breast cancer-specific survival (BCSS) after propensity score matching (PSM), and attempted to identify precise subgroups of patients that could benefit from NART.

**Results:**

1785 patients receiving NART were identified from a total of 274,199 patients between 2010 and 2021, with a median follow-up time of around 58 months. NART utilization in higher tumor stages shows a slight upward trend. Based on the multivariate logistic regression model, age more than 50 years old (OR = 0.86; 95% CI 0.78 to 0.94), stage II–III (II: OR = 1.49; 95% CI 1.28 to 1.74 III: OR = 2.44; 95% CI 1.98 to 3.00), T4 (OR = 1.52; 95% CI 1.25 to 1.84), HR-/HER2- (OR = 0.8; 95% CI 0.70 to 0.92) were independently correlated with NART. PORT was significantly associated with better survival (*P* < 0.001, HR = 0.6709; 95% CI 0.5898–0.7630 for OS) after propensity score matching (PSM). Subgroup analyses showed that for patients of HR-/HER2- subtype, AJCC stage I category or patients receiving breast-conserving surgery (BCS), NART and PORT resulted in similar OS and BCSS. We also identified risk factors for second primary malignancies development after breast cancer and found that NART were related to the lower risk of developing second primary malignancies following breast cancer.

**Conclusion:**

Over time, an increasing number of breast cancer patients received NART. We cannot affirm that NART is as effective as PORT in the treatment of BC, however we can consider NART as an alternative option in some contexts such as patients of AJCC stage I category and patients receiving BCS, given its potential advantages of reducing second primary malignancies risk.

**Supplementary Information:**

The online version contains supplementary material available at https://doi.org/10.1007/s12672-026-05466-3.

## Introduction

Breast cancer (BC) constitutes one of the most common malignancies affecting the female population [1]. As the predominant therapeutic approach in the field of oncology, radiotherapy occupies a critical position in the adjuvant treatment of BC [2]. Radiotherapy (RT), including preoperative, intraoperative, and postoperative modalities, is recognized as an effective strategy to reduce BC metastasis and improving patient survival [3, 4]. Nevertheless, the field of preoperative neoadjuvant radiotherapy (NART) remains conspicuously underexplored in clinical trials.

The standard of care for breast cancer treatment is postoperative radiotherapy following breast-conserving surgery. However, patients who undergo standard whole breast irradiation (WBI) are subjected to some radiation to the heart and lungs which may have a detrimental effect on their long-term health, with higher risks being associated with larger volumes of irradiated organs at risk [5]. As a result, techniques such as breathing-adapted image-guided RT and partial-breast irradiation (PBI) have been developed to reduce toxicity without compromising the oncologic outcomes [6]. Additionally, accelerated partial-breast irradiation (APBI) has been proposed as a strategy to improve adherence to adjuvant breast irradiation by reducing the number of required treatment visits and associated transportation burdens. Results from phase I-II clinical trials have demonstrated that preoperative PBI is feasible and well-tolerated in patients with early-stage breast cancer, accompanied by high local control rates. The potential advantages of administering PBI prior to surgery include reduced target volumes, improved treatment accuracy, a lower risk of fibrosis (attributable to the subsequent removal of the breast tissue that received the highest radiation dose), and satisfactory cosmetic outcomes [7].

NART is well established in the treatment of several types of cancer that are radiosensitive, such as esophageal cancer [8, 9], rectal cancer [10, 11], and sarcoma [12–14] and has also been combined with neoadjuvant systemic therapy to render unresectable, locally advanced breast cancers operable [15, 16]. Moreover, for patients who are both qualified for and choose surgical intervention and subsequent breast reconstruction, NART lays the groundwork for immediate reconstruction [7, 17–19]. The effectiveness of post-operative adjuvant radiotherapy (PORT) may be reduced compared to NART due to surgical tissue damage that affects the blood supply to tumor tissue [15, 20]. The resulting hypoxic state could increase the resistance of tumor cells to radiotherapy, leading to a diminished response, reflected as an increased likelihood of tumor recurrence in areas such as the chest wall. Neoadjuvant radiotherapy effectively mitigates these risks [15, 20]. Furthermore, NART could serve as a tool for treatment stratification for de-escalation of treatments in the event of pathological complete response [20, 21].

According to previous studies, there exist conflicting results on the effect of NART. The discrepancies are largely attributable to significant differences in the study populations across published reports. Poleszczuk et al.’s study analyzed patients diagnosed between 1973 and 2011 in SEER database with carcinoma in situ or localized breast cancer (T1–T3) without prior cancer, posited that NART could significantly improve disease-free survival without reducing overall survival for estrogen receptor-positive patients with early-stage breast cancer. Their analysis finally supported this assumption [22]. However, not all clinical research findings underscore the considerable advantages of NART. Fu et al. scrutinized the SEER registry of patients diagnosed with BC from 1973 to 2013 and found that patients receiving NART have significantly lower breast cancer-specific survival (BCSS) and overall survival (OS) compared with those treated with PORT [23]. These differences are mainly attributed to different effects of NART between different subgroups. In addition, Since the SEER database did not include HER2 status data before 2010, previous studies were only classified based on hormone receptor status. To date, prospective multi-center trials about the optimal timing and sequencing of RT and breast surgery are still pending [24–28]. Our study comprehensively assessed trends in the management of NART in the population during the past decade and attempted to identify precise subgroups of patients that could benefit from NART.

## Methods

### Data source and patient population

**Fig. 1:**
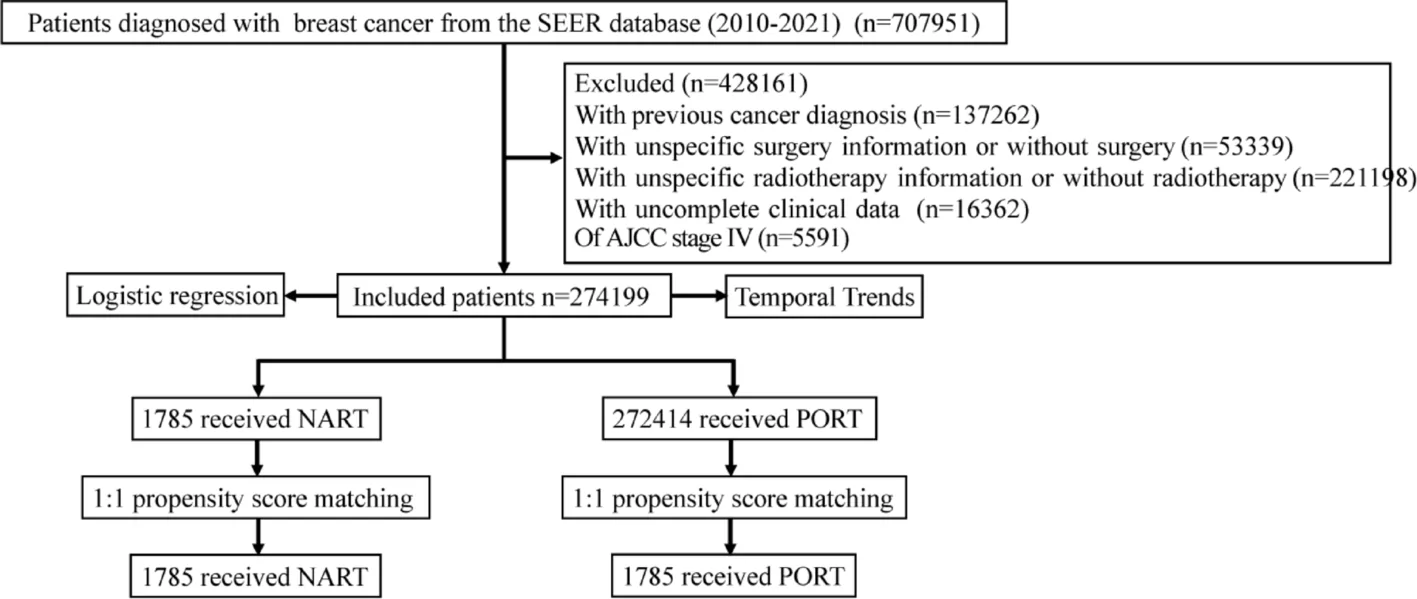
Flow diagram of study cohort selection

The database from the National Cancer Institute’s SEER program [https://seer.cancer.gov/] was queried to build our study cohort. The SEER database includes 18 registries covering approximately 28% of the U.S. population and contains basic demographics and some clinical characteristics. We used the SEER database, to include all participants diagnosed with microscopically confirmed invasive breast carcinoma between the years 2010–2021. Infiltrating ductal carcinoma histology was identified with SEER ICD-0-3 codes: 8500. All patients with insufficient demographic data, with other primary cancer before and without receiving radiotherapy or surgery were excluded from the study. The following clinicopathological factors were extracted from the SEER database: age at diagnosis; race; histologic grade; AJCC stage; molecular subtype; T stage; N stage and treatment data including surgery for the primary site, chemotherapy record, and adjuvant radiotherapy (Fig. 1).

### Propensity score matching

Propensity score matching (PSM) [29] serves as a critical statistical tool to mitigate selection bias in non-randomized observational studies, thereby achieving covariate balance between different treatment groups. The propensity score refers to the conditional probability of a study subject being allocated to a specific treatment arm, which is calculated based on a set of observed baseline covariates. The propensity score was defined as the likelihood of undergoing NART (on a scale from 0 to 1), given individual characteristics. It was obtained from a logistic regression model that factored the independent correlations of all retrieved variables (i–x) on NART status (xi). Subjects were matched by propensity score to their nearest neighbor in a 1:1 ratio without replacement. We created a matched dataset using PSM, using age (over and equal or under 50 years old), race, histologic grade, AJCC stage, molecular subtype, T stage, N stage and treatment options including surgery (breast-conserving surgery or mastectomy) and chemotherapy (yes versus no) as covariates. Then, PSM was conducted at a 1:1 matching ratio to create a matched pair between the NART group and the PORT group. Validation of PSM was achieved by comparing the NART and PORT groups for each observed variable before and after PSM.

### Statistical analysis

The Chi-square test was applied to compare categorical variables across different groups. Univariate logistic regression analysis was performed, and variables with *P* < 0.05 were included in the multivariate logistic regression model. Separate multivariable logistic regression models were utilized to screen out factors that were independently associated with the receipt of non-adjuvant radiotherapy (NART) (*P* < 0.5). Univariate and multivariate Cox proportional hazard models were employed to identify factors correlated with improved overall survival (OS) and breast cancer-specific survival (BCSS), and the results were reported as hazard ratio (HR) along with 95% confidence intervals (CI). For breast cancer specific survival (BCSS), only deaths caused by breast cancer were considered events, while for overall survival (OS), deaths caused by any reason were regarded as events. The SEER database records the causes of death of patients in detail. We excluded patients who died of other causes, thereby identifying cases of death caused by breast cancer. The Kaplan–Meier method was used to estimate survival curves, and the log-rank test was conducted to compare survival outcomes between groups classified by nominal variables. All statistical analyses were implemented using R software (version 4.0.2), and a two-sided p value < 0.05 was considered statistically significant.

## Results

### Patient characteristics

A total of 707951 patients with breast cancer without prior cancer elsewhere were enrolled. After excluding the patients with nonmatching data, 274199 patients remained in the final cohort. We divided the patients into two distinct groups, those who received radiotherapy before surgery (NART, *n* = 1785) and those who received radiotherapy after surgery (PORT, *n* = 272414). Patients without surgery or systemic therapy were excluded from the study (Fig. 1). The baseline characteristics of patients with breast cancer by treatment modality are shown in Table 1. Among patients receiving NART, a higher proportion of older patients, AJCC I and HR+/HER2- disease were observed. Patients who underwent NART were more likely to receive chemotherapy (70.1% in NART and 29.9% in PORT) and BCS (57.1% in NART and 42.9% in PORT). At baseline, all variables were found to be significantly different across groups (both *p* < 0.05)). Then 1:1 PSM with a caliper of 0.1 was performed. After matching, 3570 cases were finally included in the analysis, with 1785 cases equally in each group. There was no significant difference after matching in general clinical characteristics between the two groups (all *p* > 0.05).

**Table 1:** Baseline Characteristics of Patients before and after PSM by Treatment Modality

Variables	Number of patients (%)			Number of patients (%)		
	NART	PORT	*P *value	NART	PORT	*P *value
	(*N* = 1785)	(*N* = 272414)		(*N* = 1785)	(*N* = 1785)	
Age at diagnosis			< 0.001			1
<=50	573 (32.1%)	57,901 (21.3%)		573 (32.1%)	574 (32.2%)	
> 50	1212 (67.9%)	214,513 (78.7%)		1212 (67.9%)	1211 (67.8%)	
Race			< 0.001			1
Black	245 (13.7%)	28,023 (10.3%)		245 (13.7%)	246 (13.8%)	
Other	208 (11.7%)	29,756 (10.9%)		208 (11.7%)	209 (11.7%)	
White	1332 (74.6%)	214,635 (78.8%)		1332 (74.6%)	1330 (74.5%)	
Histologic grade			< 0.001			1
I	139 (7.8%)	39,819 (14.6%)		139 (7.8%)	138 (7.7%)	
II	421 (23.6%)	73,976 (27.2%)		421 (23.6%)	422 (23.6%)	
III	484 (27.1%)	48,764 (17.9%)		484 (27.1%)	486 (27.2%)	
Unknown	741 (41.5%)	109,855 (40.3%)		741 (41.5%)	739 (41.4%)	
AJCC stage			< 0.001			1
I	675 (37.8%)	166,769 (61.2%)		675 (37.8%)	677 (37.9%)	
II	634 (35.5%)	74,370 (27.3%)		634 (35.5%)	632 (35.4%)	
III	476 (26.7%)	31,275 (11.5%)		476 (26.7%)	476 (26.7%)	
T			< 0.001			1
T1	715 (40.1%)	169,000 (62.0%)		715 (40.1%)	711 (39.8%)	
T2	690 (38.7%)	80,114 (29.4%)		690 (38.7%)	691 (38.7%)	
T3	206 (11.5%)	17,124 (6.3%)		206 (11.5%)	209 (11.7%)	
T4	174 (9.7%)	6176 (2.3%)		174 (9.7%)	174 (9.7%)	
N			< 0.001			1
N0	872 (48.9%)	181,587 (66.7%)		872 (48.9%)	871 (48.8%)	
N1	654 (36.6%)	67,923 (24.9%)		654 (36.6%)	657 (36.8%)	
N2	153 (8.6%)	14,665 (5.4%)		153 (8.6%)	151 (8.5%)	
N3	106 (5.9%)	8239 (3.0%)		106 (5.9%)	106 (5.9%)	
Subtype			< 0.001			1
HR-/HER2-	313 (17.5%)	26,794 (9.8%)		313 (17.5%)	315 (17.6%)	
HR-/HER2+	135 (7.6%)	9923 (3.6%)		135 (7.6%)	135 (7.6%)	
HR+/HER2-	1066 (59.7%)	208,815 (76.7%)		1066 (59.7%)	1067 (59.8%)	
HR+/HER2+	271 (15.2%)	26,882 (9.9%)		271 (15.2%)	268 (15.0%)	
Surgery			< 0.001			0.8
BCS	1019 (57.1%)	216,888 (79.6%)		1019 (57.1%)	1026 (57.5%)	
Mastectomy	766 (42.9%)	55,526 (20.4%)		766 (42.9%)	759 (42.5%)	
Chemotherapy			< 0.001			0.8
No/Unknown	534 (29.9%)	152,476 (56.0%)		534 (29.9%)	527 (29.5%)	
Yes	1251 (70.1%)	119,938 (44.0%)		1251 (70.1%)	1258 (70.5%)	

### Factors associated with neoadjuvant radiotherapy

As the significance of neoadjuvant radiotherapy for breast cancer was still somewhat controversial, the factors associated with neoadjuvant radiotherapy among breast cancer were then explored (before PSM). Variables with statistically significant difference (*P* < 0.05) in the univariate logistic regression associated with neoadjuvant radiotherapy were younger age, black race, higher histologic grade or tumor stage and chemotherapy (Table 2). Age < = 50, higher histologic grade, higher AJCC stage, lager tumor size and ER- subtype were correlated with NART in the univariate regression. Based on the multivariate logistic regression model, age less than 50 years old (OR = 0.83; 95% CI 0.75 to 0.92), AJCC stage II–III (II: OR = 1.35; 95% CI 1.15 to 1.59 III: OR = 1.41; 95% CI 1.14 to 1.75), T4 (OR = 2.36; 95% CI 1.86 to 3.00) and ER- subtype (OR = 0.75; 95% CI 0.67 to 0.85) were independently correlated with NART (Table 2).

**Table 2:** Univariate and multivariate logistic regression of factors associated with NART

Variables	Univariate logistic regression			Multivariate logistic regression		
	OR	95% CI	*P* value	OR	95% CI	*P* value
Age at diagnosis						
Age > 50 vs. <=50	0.57	0.52–0.63	< 0.001	0.83	0.75–0.92	< 0.001
Race						
Other vs. black	0.80	0.66–0.96	0.018	0.92	0.77–1.12	0.412
White vs. black	0.71	0.62–0.81	< 0.001	0.91	0.79–1.05	0.190
Histologic grade						
II vs. I	1.63	1.35–1.98	< 0.001	1.10	0.91–1.34	0.329
III vs. I	2.84	2.35–3.44	< 0.001	1.21	0.98–1.48	0.073
Unknown vs. I	1.93	1.61–2.32	< 0.001	1.30	1.07–1.57	0.007
AJCC stage						
AJCC.II vs. I	2.11	1.89–2.35	< 0.001	1.35	1.15–1.59	< 0.001
AJCC.III vs. I	3.76	3.34–4.23	< 0.001	1.41	1.14–1.75	0.002
T						
T2 vs. T1	2.04	1.83–2.26	< 0.001	1.15	1.00-1.32	0.055
T3 vs. T1	2.84	2.43–3.32	< 0.001	1.18	0.96–1.44	0.115
T4 vs. T1	6.66	5.63–7.87	< 0.001	2.36	1.86-3.00	< 0.001
N						
N1-N3 vs. N0	2.09	1.91–2.30	< 0.001	0.90	0.79–1.02	0.109
Chemotherapy						
Yes vs. No/Unknown	2.98	2.69–3.30	< 0.001	1.56	1.36–1.78	< 0.001
ER status						
ER + vs. ER-	0.46	0.42–0.52	< 0.001	0.75	0.67–0.85	< 0.001
Surgery						
Mastectomy vs. BCS	2.94	2.67–3.23	< 0.001	1.66	1.46–1.89	< 0.001

### Survival analysis in propensity score-matched cohort

Due to the significant differences in clinical-pathological characteristics between NART and PORT, a 1:1 propensity score matching (PSM) was conducted successfully for the purpose of balancing (Additional file 1: Fig. S1). After the 1:1 case control matching by PSM, 1785 NART cases were matched with 1785 PORT cases (Table 1). In the matched data, older age, higher grade, advanced T stage, advanced N stage, advanced AJCC stage, HR-/HER2- and NART were all identified as risk factors for overall survival (OS). PORT was significantly associated with better survival after multivariate analysis (*P* < 0.001, HR = 0.618; 95% CI 0.522–0.731 for OS) compared to those who received NART (Additional file 1: Fig. S2). In addition, higher mortality rates were independently correlated with being over the age of 50, having a higher histologic grade, larger tumor size, and having a pathological N category of N2 or higher (Table 3). We also performed multivariate Cox regression without PSM, which showed consistent outcomes (Table S1).

**Table 3:** Univariate and multivariate Cox regression analysis for overall survival in the matched data

Variables	Univariate analysis			Multivariate analysis		
	HR	95% CI	*P* value	HR	95% CI	*P* value
Age at diagnosis						
Age > 50 vs. <=50	1.09	0.915–1.31	0.32	1.274	1.055.-1.538	0.012
Race						
Other vs. black	0.599	0.425–0.842	< 0.01	0.727	0.513–1.031	0.074
White vs. black	0.712	0.572–0.885	< 0.01	0.862	0.689–1.079	0.195
Histologic grade						
II vs. I	1.79	1.18–2.72	0.006	1.476	0.966–2.255	0.072
III vs. I	3.47	2.32–5.19	< 0.001	2.292	1.500-3.502	< 0.001
Unknown vs. I	2.50	1.62–3.84	< 0.001	2.087	1.343–3.241	0.001
AJCC stage						
AJCC.II vs. I	2.17	1.64–2.88	< 0.001	1.416	0.961.-2.086	0.079
AJCC.III vs. I	5.91	4.54–7.69	< 0.001	1.722	1.067–2.781	0.026
T						
T2 vs. T1	2.13	1.68–2.69	< 0.001	1.543	1.146–2.078	0.004
T3 vs. T1	3.71	2.84–4.86	< 0.001	2.095	1.463-3.000	< 0.001
T4 vs. T1	6.30	4.90–8.10	< 0.001	2.849	1.952–4.158	< 0.001
N						
N1 vs. N0	2.01	1.64–2.47	< 0.001	1.445	1.131–1.846	0.003
N2 vs. N0	3.64	2.83–4.66	< 0.001	2.025	1.434–2.857	< 0.001
N3 vs. N0	4.41	3.37–5.77	< 0.001	2.169	1.519–3.098.	< 0.001
Chemotherapy						
Yes vs. No/Unknown	1.8	1.45–2.22	< 0.001	0.618	0.477–0.801	< 0.001
Subtype						
HR-/HER2 + vs. HR-/HER2-	0.430	0.304–0.610	< 0.001	0.373	0.262–0.531	< 0.001
HR+/HER2- vs. HR-/HER2-	0.449	0.372–0.543	< 0.001	0.560	0.452–0.694	< 0.001
HR+/HER2 + vs.HR-/HER2-	0.329	0.244–0.444	< 0.001	0.374	0.275–0.509	< 0.001
Surgery						
Mastectomy vs. BCS	2.59	2.17–3.08	< 0.001	1.373	1.105–1.706	0.004
Radiotherapy						
PORT vs. NART	0.662	0.56–0.783	< 0.001	0.618	0.522–0.731	< 0.001

### Subgroup interaction analysis

**Fig. 2:**
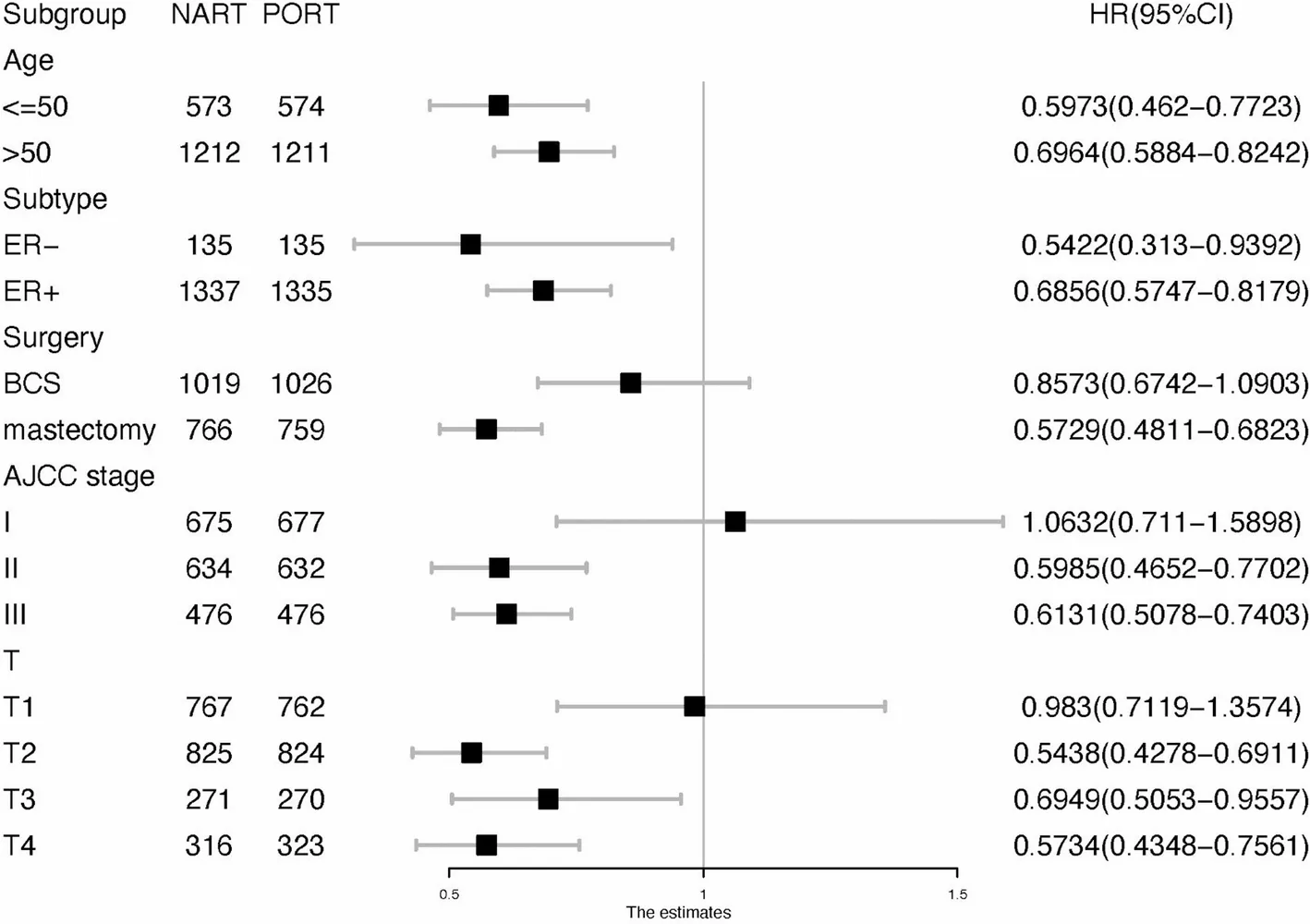
Forest plot of the association of neoadjuvant radiotherapy (NART) with overall survival in subgroup analyses

To further identify patients who may benefit from NART, subgroup analyses were performed after PSM. We obtained an HR in each category comparing PORT to NART (Fig. 2). NART was associated with worse prognosis in most of these categories. However, patients of AJCC stage I category (HR = 1.0632; 95% CI 0.711 to 1.5898), pathological T1 category (HR = 0.983; 95% CI 0.712 to 1.357) or patients receiving BCS (HR = 0.8573; 95% CI 0.6742 to 1.0903) showed no significant difference in overall survival, which implied that these patients could take consideration of NART in clinical practice (Fig. 2).

**Fig. 3:**
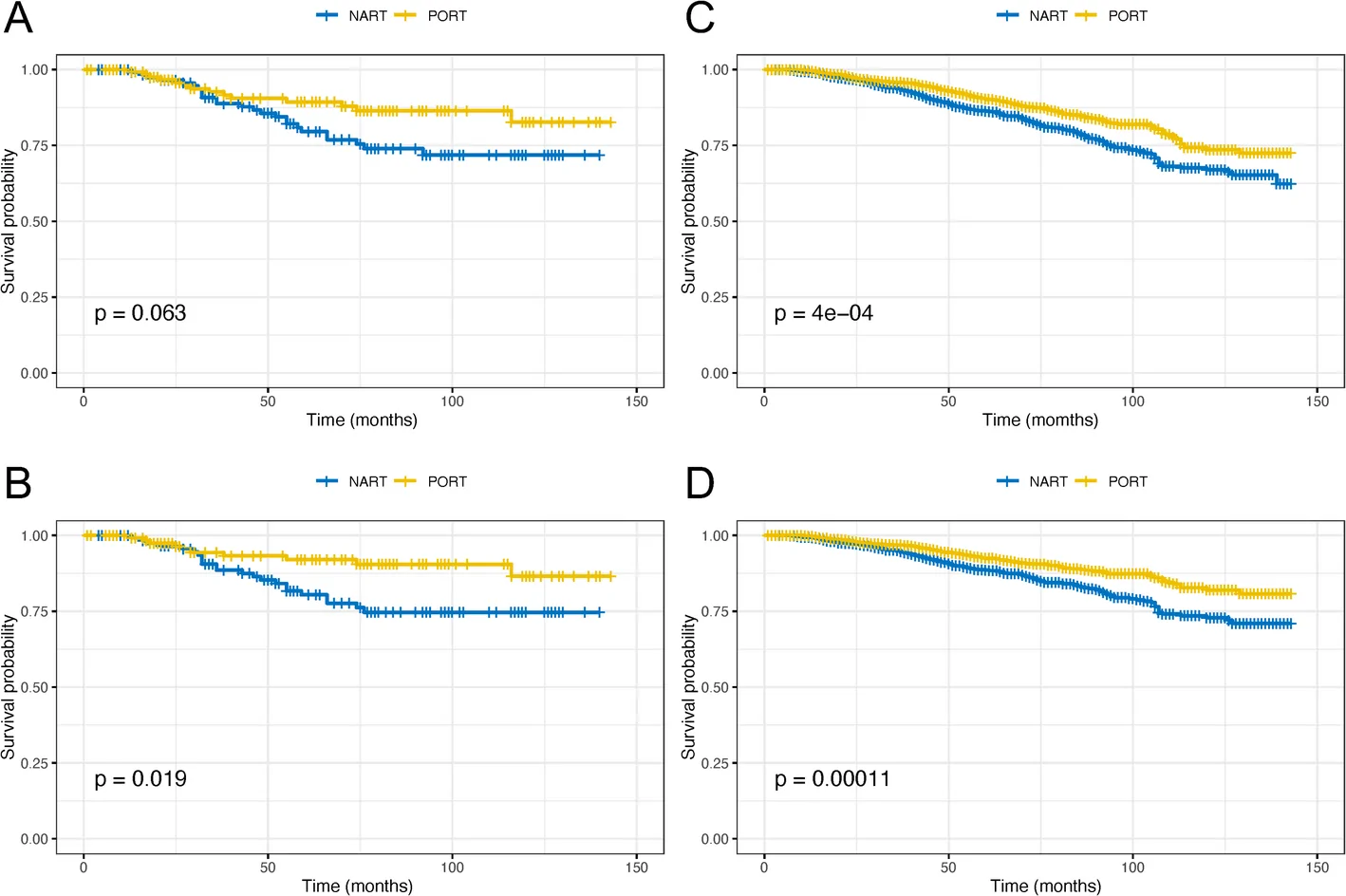
Subgroup analyses of Kaplan–Meier survival curves of OS and BCSS based on molecular subtype stratified by radiotherapy types. **A** KM curves of OS in ER- subtype; **B** KM curves of BCSS in ER- subtype; **C** KM curves of OS in ER+ subtype; **D** KM curves of BCSS in ER+ subtype

**Fig. 4:**
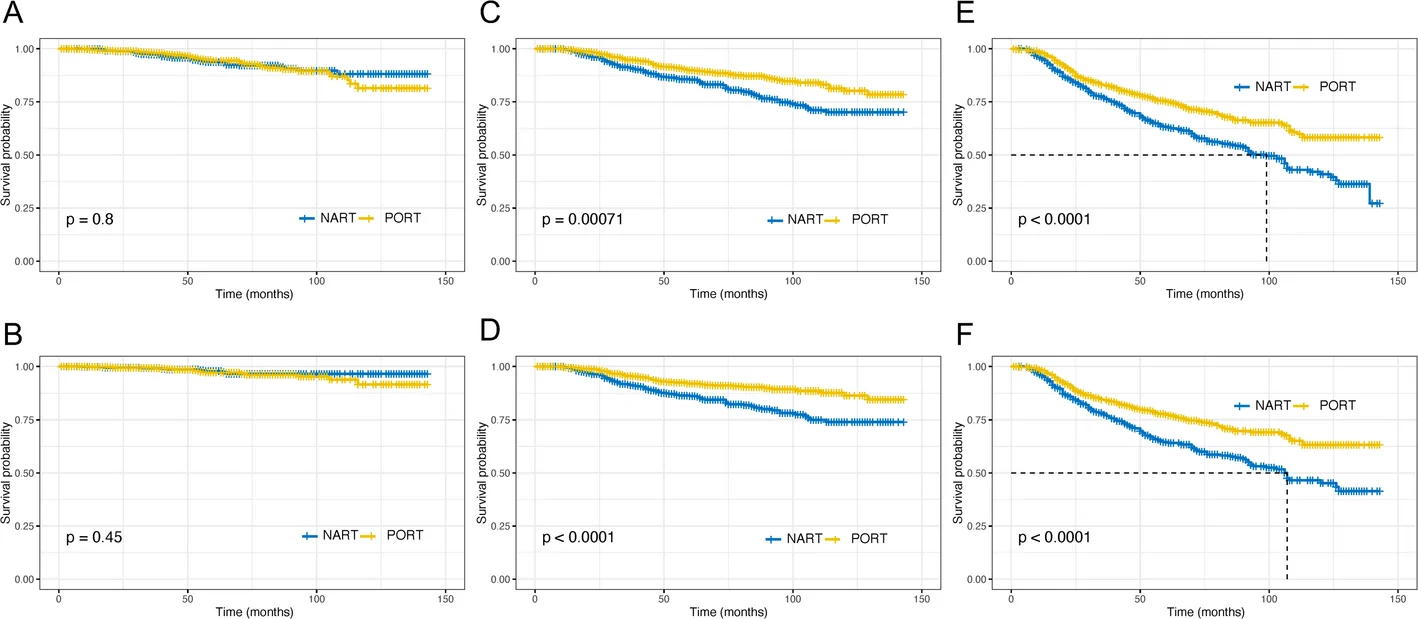
Subgroup analyses of Kaplan–Meier survival curves of OS and BCSS based on tumor stage stratified by radiotherapy types. **A** KM curves of OS in stage I; **B** KM curves of BCSS in stage I; **C** KM curves of OS in stage II; **D** KM curves of BCSS in stage II; **E** KM curves of OS in stage III; **F** KM curves of BCSS in stage III

**Fig. 5:**
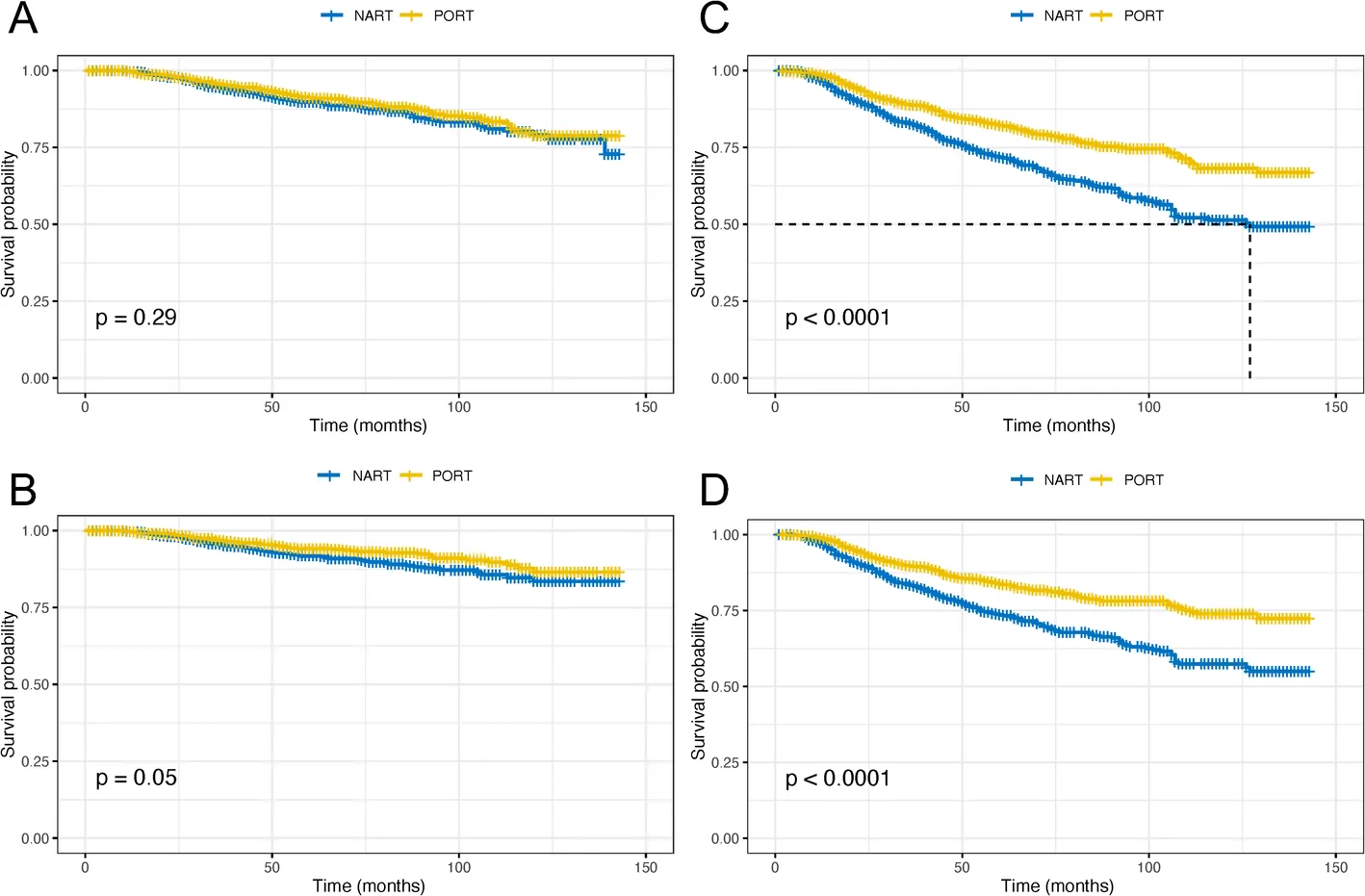
Subgroup analyses of Kaplan–Meier survival curves of OS and BCSS based on surgical procedure stratified by radiotherapy types. **A** KM curves of OS in patients receiving BCS; **B** KM curves of BCSS in patients receiving BCS; **C** KM curves of OS in patients receiving mastectomy; **D** KM curves of BCSS in patients receiving mastectomy

Based on the matched cohort, the OS and BCSS outcomes remained no significant difference between PORT and NART in patients of AJCC stage I groups and patients undergoing BCS (Figs. 4A and B and 5A and B; *p* > 0.05). While ER+ patients, patients of II-III stages and patients undergoing mastectomy showed longer OS and CSS from PORT compared to NART (Figs. 4C–H and 5C and D; *P* < 0.01). ER- patients showed similar OS while different BCSS between PORT and NART (Fig. 3A-D).

**Fig. 6:**
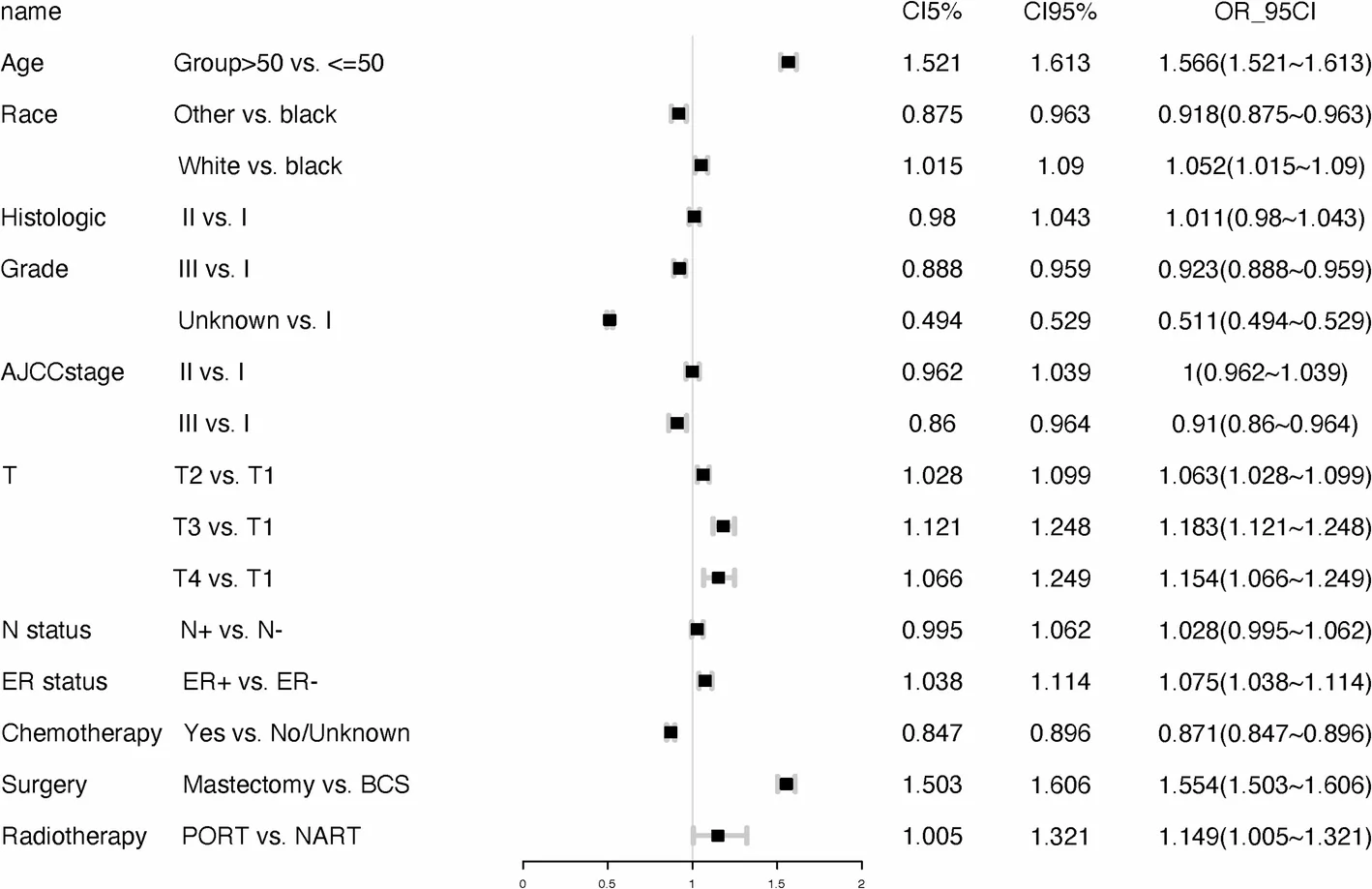
Multivariable logistic regression analysis of risk of developing second primary malignancies

We also analyzed the risk of developing second primary malignancies. Patients treated with NART (HR 1.149, 95% CI 1.005–1.321, *p* < 0.05) had a lower risk of developing second primary malignancies after BC (Fig. 6).

## Discussion

There are no well-established guidelines for NART. The benefits of RT prior to surgical treatment are not conclusive and predictive biomarkers of response are unknown [30]. Currently, only a few studies directly compared the survival between neoadjuvant and adjuvant RT in patients with BC disease. Compared with previous works, our study is the first to include breast cancer data from the past decade and conduct a comprehensive analysis, including factors related to neoadjuvant radiotherapy and prognostic analysis of different subgroups. Our study has certain strengths and implications for clinical practice. With this study we cannot affirm that NART is as effective as PORT in the treatment of BC, but we can consider NART as an alternative option in some contexts such as patients of AJCC stage I category and patients receiving BCS. This is probably because neoadjuvant radiotherapy can reduce the risk of developing second primary malignancies.

In the present study, the overall prognosis of the NART group was inferior to that of the PORT group, which is probably attributed to the inherent selection bias inherent in retrospective observational studies. No information regarding selection criteria including systematic therapy information and radiation protocols (total dose, fractionation) for neoadjuvant RT was recorded in the SEER database. Although Propensity score matching (PSM) was employed to balance baseline characteristics between the two groups, this inherent selection bias cannot be completely eliminated. Even so, we recommend NART as an alternative option for a specifically selected subgroup (defined in the present study as AJCC stage I disease, and those who underwent breast-conserving surgery), considering its potential advantages of reducing second primary malignancies risk. Within these subgroups, there were no statistically significant difference in survival benefit between NART and PORT. Furthermore, NART offers unique clinical advantages: better tumor volume identification, possible tumor downstaging with improved rates of breast preservation rates, improved cosmesis, shortening of treatment time, reduction of complication rates in patients requiring breast reconstruction.

Traditionally, the effectiveness of postoperative adjuvant radiotherapy can be reduced due to surgical tissue damage that affects the blood supply to tumor tissue. The resulting hypoxic state could increase the resistance of tumor cells to radiotherapy, leading to a reduced response and an increased likelihood of tumor recurrence in areas such as the chest wall. Pre-operative radiotherapy effectively reduces these risks [15, 31]. However, it should be recognized that preoperative NART may induce excessive fibrosis of tumor tissue and blood vessels, which may make it difficult to separate tumor tissue during surgery [15, 24]. It will be important to carefully regulate the intensity and duration of radiation during preoperative NART to minimize or mitigate any potential adverse effects on surgical procedures.

As one of the most promising modalities of radiotherapy, stereotactic body radiotherapy (SBRT) involves the delivery of a very high dose of radiation to the tumor volume with high precision using one or several fractions administered usually in 1 to 10 days [32]. The biological efficacy of SBRT is not only based on the mechanism of DNA damage at the molecular level, but also on additional effects at the tissue and cellular level (damaging the vasculature and cell membranes), as well as on the abscopal effect of enhancing the anti-tumor immune response due to the action of high doses of radiotherapy on the cancerous tumor, which produces a therapeutic effect beyond the irradiated field [33, 34]. Notable is the lack of a standardized radiotherapy dose and time interval between the end of radiotherapy and the performance of surgery. The pCR rate following preoperative SBRT seems to be positively correlated with the duration between radiotherapy and surgery in a cohort of early-stage breast cancer patients possessing favorable prognostic factors. Vasmel JE et al. [35, 36] reported 33% pCR after 6 months, and 48% pCR after 8 months from radiotherapy to BCS among early-stage breast cancer patients with favorable risk factors. PCR was achieved in less than 10% of cases in studies where the time gap from the end of radiotherapy to surgery was less than 13 weeks [37–39].

Interesting studies have been published with encouraging findings both in early-stage BC and locally advanced breast cancer (LABC). Researchers in the Netherlands conducted a prospective single-arm (ABLATIVE) study of 36 patients between 2015 and 2018 of single-fraction MRI-guided preoperative SBRT for women with negative sentinel nodes and favorable non-lobular unifocal tumors up to 2 cm (age, 50–70 years) or up to 3 cm (for patients over 70 years) [35]. The primary outcome was rate of pCR, and 36 patients received 20 Gy to the gross tumor volume (GTV) and 15 Gy to the clinical target volume (CTV) followed by surgery 6 or 8 months after radiation. Neoadjuvant endocrine therapy was allowed, and 6 patients received this treatment. A pCR was seen in 15 patients [42%, 95% confidence interval (CI): 26–59%], and no patients experienced a local recurrence after a median follow-up of 21 months. All patients experienced grade 1 fibrosis in the treated breast, and transient grade 2 toxicity was observed in 31% of patients [35]. Given these favorable results, the authors concluded that preoperative SBRT with or without endocrine therapy represents a feasible treatment approach and may even allow for omission of surgery in select patients if pCR can be accurately predicted. Jasmina Mladenovic et al. reported results of 134 patients diagnosed with locally advanced breast cancer (LABC) who received NART; the radiotherapy regimen included a total radiation dose of 45 Gy, delivered in 15 fractions over a 6-week period, with the radiation targeted at the breast and regional lymph nodes. Radical mastectomy was performed 6 weeks after finishing NART treatment. Adjuvant systemic therapy was administered as established clinical protocol. pCR in the breast was detected in 15% of the enrolled patients, 7.5% of which with lymph node pCR as well. Relapses were confirmed in 61.9% of the patients and 95% of these cases were identified as distant metastasis. The 5-year DFS and 5-year OS were 39.2% and 55.1%, respectively. This study demonstrated that patients achieving clinical complete responses had a longer OS (*p* = 0.038), there was a tendency toward prolonged DFS in patients who achieved pCR following NART administration [40].

Furthermore, recent studies have indicated that radiotherapy (RT) possesses potent immunomodulatory properties, which can induce a synergistic anti-tumor effect when combined with immunotherapy and chemotherapy [16, 41]. Chen et al. published the efficacy and safety of neoadjuvant stereotactic body radiotherapy (SBRT) in combination with adebrelimab, nab-paclitaxel, and carboplatin among patients with triple-negative breast cancer (TNBC). This novel therapeutic strategy exhibited favorable anti-tumor activity, achieving a pathological complete response (pCR) rate of 90%, along with a residual cancer burden (RCB) 0-I rate and an objective response rate (ORR) of 100% respectively [42]. However, no further subgroup analysis was conducted in this study. In addition, the sample sizes of the two studies were relatively small, which may have introduced potential bias.

There are several limitations to our study. Unmeasured confounders such as pCR following NART and systematic treatment were not available in the SEER database and may have influenced overall results. When subgroup analyses were performed according to surgical procedures, undergoing breast-conserving surgery means patients may had already benefited from neoadjuvant chemotherapy or targeted therapy. Second, the detailed regimes for systematic treatment and radiation protocols (total dose, fractionation) are not provided in the SEER database, which may lead to a potential bias in determining the role of NART in BC patients. Finally, because this was a retrospective study, there may have been selection bias. These questions need to be addressed by further prospective randomized controlled trials. Prospective studies should be developed to evaluate tumors and patient characteristics in order to identify predictive response factors and promote accuracy in patient selection.

## Supplementary Information

Below is the link to the electronic supplementary material.


Additional file 1.


## Data Availability

The datasets generated and analyzed during the current study are publicly available in Surveillance, Epidemiology, and End Results (SEER) database [https://seer.cancer.gov/].

## Abbreviations

BC: Breast cancer
NART: Neoadjuvant radiotherapy
PORT: Postoperative adjuvant radiotherapy
SEER: Surveillance, Epidemiology, and End Results
OS: Overall survival
BCSS: Breast cancer-specific survival
PSM: Propensity score matching
BCS: Breast-conserving surgery
pCR: pathological complete response
TME: Tumor microenvironment
HR: Hazard ratio
CI: Confidence interval
OR: Odds Ratio
AJCC: American Joint Committee on Cancer
SBRT: Stereotactic body radiotherapy
