# Supplementary material for: Evaluating the value of neoadjuvant radiotherapy in patients with breast cancer
Source: Discov Oncol. 2026 Jun 20;17:1272. doi: 10.1007/s12672-026-05466-3 (PMC13530056; doi:10.1007/s12672-026-05466-3)
Supplement: Supplementary file 1 [file 12672_2026_5466_MOESM1_ESM.docx]

**Supplementary figures**

**Figure S1.** Distribution and histograms of propensity scores before and after matching.

**Figure S2.** Overall survival (A) and Breast cancer-specific survival (B) stratified by adjuvant radiotherapy in all matched patients after PSM.

| **Variables** | **Univariate analysis** | | |
| --- | --- | --- | --- |
|  | **HR** | **95% CI** | **P value** |
| **Age at diagnosis** |  |  |  |
| Age>50 vs. <=50 | 1.747 | 1.689-1.807 | <.001 |
| **Race** |  |  |  |
| Other vs. black | 0.574 | 0.544-0.607 | <.001 |
| White vs. black | 0.764 | 0.738-0.791 | <.001 |
| **Histologic grade** |  |  |  |
| II vs. I | 1.218 | 1.171-1.266 | <.001 |
| III vs. I | 1.696 | 1.624-1.772 | <.001 |
| Unknown vs. I | 1.325 | 1.263-1.391 | <.001 |
| **AJCC stage** |  |  |  |
| AJCC.II vs. I | 1.302 | 1.239-1.369 | <.001 |
| AJCC.III vs. I | 1.589 | 1.467-1.721 | <.001 |
| **T** |  |  |  |
| T2 vs. T1 | 1.482 | 1.422-1.544 | <.001 |
| T3 vs. T1 | 1.793 | 1.691-1.900 | <.001 |
| T4 vs. T1 | 2.765 | 2.581-2.961 | <.001 |
| **N** |  |  |  |
| N1 vs. N0 | 1.365 | 1.312-1.419 | <.001 |
| N2 vs. N0 | 1.975 | 1.844-2.115 | <.001 |
| N3 vs. N0 | 2.911 | 2.713-3.124 | <.001 |
| **Chemotherapy** |  |  |  |
| Yes vs. No/Unknown | 0.531 | 0.513-0.549 | <.001 |
| **Subtype** |  |  |  |
| HR-/HER2+ vs. HR-/HER2- | 0.504 | 0.472-0.537 | <.001 |
| HR+/HER2- vs. HR-/HER2- | 0.513 | 0.494-0.532 | <.001 |
| HR+/HER2+vs.HR-/HER2- | 0.440 | 0.418-0.462 | <.001 |
| **Surgery** |  |  |  |
| Mastectomy vs. BCS | 1.275 | 1.231-1.320 | <.001 |
| **Radiotherapy** |  |  |  |
| PORT vs. NART | 0.633 | 0.568-0.706 | <.001 |

**Table. S1** Multivariate Cox regression analysis without PSM
